# Simultaneous exposure to FcγR and FcαR on monocytes and macrophages enhances antitumor activity *in vivo*

**DOI:** 10.18632/oncotarget.17000

**Published:** 2017-04-10

**Authors:** Bingyu Li, Lijun Xu, Fei Tao, Kun Xie, Zhiqiang Wu, You Li, Jie Li, Kaiming Chen, Chenyu Pi, Andrew Mendelsohn, James W. Larrick, Hua Gu, Jianmin Fang

**Affiliations:** ^1^ School of Life Sciences and Technology, Tongji University, Shanghai, China; ^2^ Panorama Research Institute, Sunnyvale, CA, USA; ^3^ Shanghai Tongji Hospital, Tongji University, Shanghai, China; ^4^ Tongji University Suzhou Institute, Suzhou, China; ^5^ Collaborative Innovation Center for Biotherapy, West China Hospital, Sichuan University, Chengdu, China; ^6^ Shanghai Key Laboratory of Signaling and Disease Research, School of Life Sciences and Technology, Tongji University, Shanghai, China

**Keywords:** antitumor activity, relapse, antibody-dependent cell-mediated cytotoxicity, in vivo mouse model, CD20

## Abstract

Therapeutic antibodies are effective for tumor immunotherapy and exhibit prominent clinical effects. All approved antibody therapeutics utilize IgG as the molecular format. Antibody-dependent cell-mediated cytotoxicity (ADCC) is a key mechanism for tumor cell killing by antibodies. For IgG antibodies, ADCC depends on FcγR-expressing cells, such as natural killer (NK) cells. However, in patients with a high tumor burden, antibody therapeutics may lose efficacy owing to exhaustion of FcγR-expressing effector cells as well as the inhibitory effects of certain FcγRs on effector cells. To achieve more potent effector functions, we engineered an anti-CD20 antibody to contain both IgG Fc and IgA Fc domains. These engineered antibodies interacted with both IgG and IgA Fc receptors (FcγR and FcαR) and recruited a broader range of effector cells, including monocytes, macrophages, neutrophils, and NK cells, thereby enhancing antibody-dependent cellular phagocytosis. Using transgenic mice expressing the FcαRI (CD89) in macrophages, we demonstrated that recombinant antibodies bearing the chimeric IgG and IgA Fc exhibited potent *in vivo* antitumor activity. Additionally, in a short-term peritoneal model using CD20-transfected LLC target cells, the *in vivo* cytotoxic activity of hybrid recombinant antibodies was mediated by macrophages with significant reduction in the absence of FcαRI. Our findings supported targeting of FcαRI on monocytes and macrophages for improved tumor immunotherapy.

## INTRODUCTION

CD20 is a glycosylated phosphoprotein expressed on most mature B-cells and a clinically validated target for non-Hodgkin's lymphoma (NHL) and autoimmune diseases. Rituximab was the first anti-CD20 monoclonal antibody approved by the US Food and Drug Administration (FDA) for the treatment of patients with B-cell lymphomas [1]. Typically, anticancer monoclonal antibodies (mAbs) mediate antitumor effects via a variety of mechanisms, including cell cycle arrest signals, direct induction of apoptosis, and sensitization to cytotoxic drugs [2]. Antibody-dependent cell-mediated cytotoxicity (ADCC) and complement-mediated cytotoxicity (CDC) are additional important mechanisms in tumor therapy [2]. All clinically approved antitumor antibodies have the IgG isotype because of their long half-life, and technologies have been established for large-scale production and purification.

IgA, the most abundant antibody class found on mucosal surfaces, plays an important role in the immune system [2]. FcαRI (CD89) is the most important IgA receptor expressed on polymorphonuclear cells (PMNs), monocytes, macrophages, and Kupffer cells [3]. FcαRI plays an important role in triggering various immunological effector functions, including induction of an oxidative burst, phagocytosis, and ADCC [2].

IgA antitumor antibodies have been shown to mediate efficient tumor lysis both *in vitro* [2] and *in vivo* using human FcαRI transgenic (Tg) mice [2]. However, daily injections of IgA antibodies are needed to achieve ideal antitumor effects *in vivo* due to the short half-life (only 15 h) of IgA in mice. Moreover, a tandem molecule containing IgG1 and IgA2, which has a half-life similar to that of IgG, has been shown to exhibit more potent antitumor activity, regulating natural killer (NK) cell- or PMN-mediated ADCC and macrophage-mediated antibody-dependent cell-mediated phagocytosis (ADCP), as described by Borrok et al [2]. However, the antitumor activity of IgG1/IgA2 antibodies has not been evaluated *in vivo*.

The innate mononuclear phagocyte network is the predominant effector cell population utilized by rituximab (anti-CD20) *in vivo* [2]. In histological sections of tumors, tumor-associated macrophages constitute the major proportion of the leukocyte tumor infiltrate, particularly for solid tumors [2]. *In vitro* studies have also found that both tumor-killing M1 and tumor-helper M2 macrophages are able to kill tumor cells in the presence of rituximab [2]. Additionally, IgA anti-epidermal growth factor receptor (EGFR) antibodies induce potent antitumor activity via M0, M1, and M2 macrophages [2].

In this study, we investigated the *in vivo* antitumor effects of an anti- CD20-IgG/IgA molecule using transgenic mice expressing CD89 on monocytes and macrophages. Our results establish a unique model to study the *in vivo* interactions of both IgG and IgA Fc with mononuclear phagocytes and have implications in the improved treatment of solid tumors.

## RESULTS

### Anti-CD20 antibodies mediated ADCC by mouse effector cells *in vitro*

To assess the antitumor activity of anti-CD20 antibodies, we previously generated CD20-IgGA by fusing the IgA2-CH_2_-CH_3_ region to rituximab (CD20-IgG) with the (G_4_S)_3_ linker. For CD20-IgA, the variable region of rituximab was fused to the IgA2m (1) constant region (Supplementary Figure 1). Rituximab was used as a CD20-IgG control. Consistent with Her2-IgGA, which was constructed in another study [2], CD20-IgGA and CD20-IgA were confirmed to have similar target affinity (Supplementary Table 1) and were as potent as rituximab in eliciting Fab-mediated antitumor effects because both constructs shared the same variable region. The antibodies were produced by transient transfection of HEK293F cells with similar levels of expression level exhibited by both CD20-IgG and CD20- IgGA constructs (Supplementary Figure 1). In accordance with previous findings, CD20-IgGA could induce ADCC mediated by either peripheral blood mononuclear cells (PBMCs) or PMNs [2]. Tumor lysis by PBMCs, which include NK cells, was only efficient with CD20-IgG. Notably, PMNs are the only blood-resident effector cells that mediate CD20-IgA activity [2]. CD20-IgGA exhibited stronger ADCC activity than CD20-IgG and CD20-IgA using human whole blood (PBMCs and PMNs) as effectors (Supplementary Figure 2).

**Figure 1:**
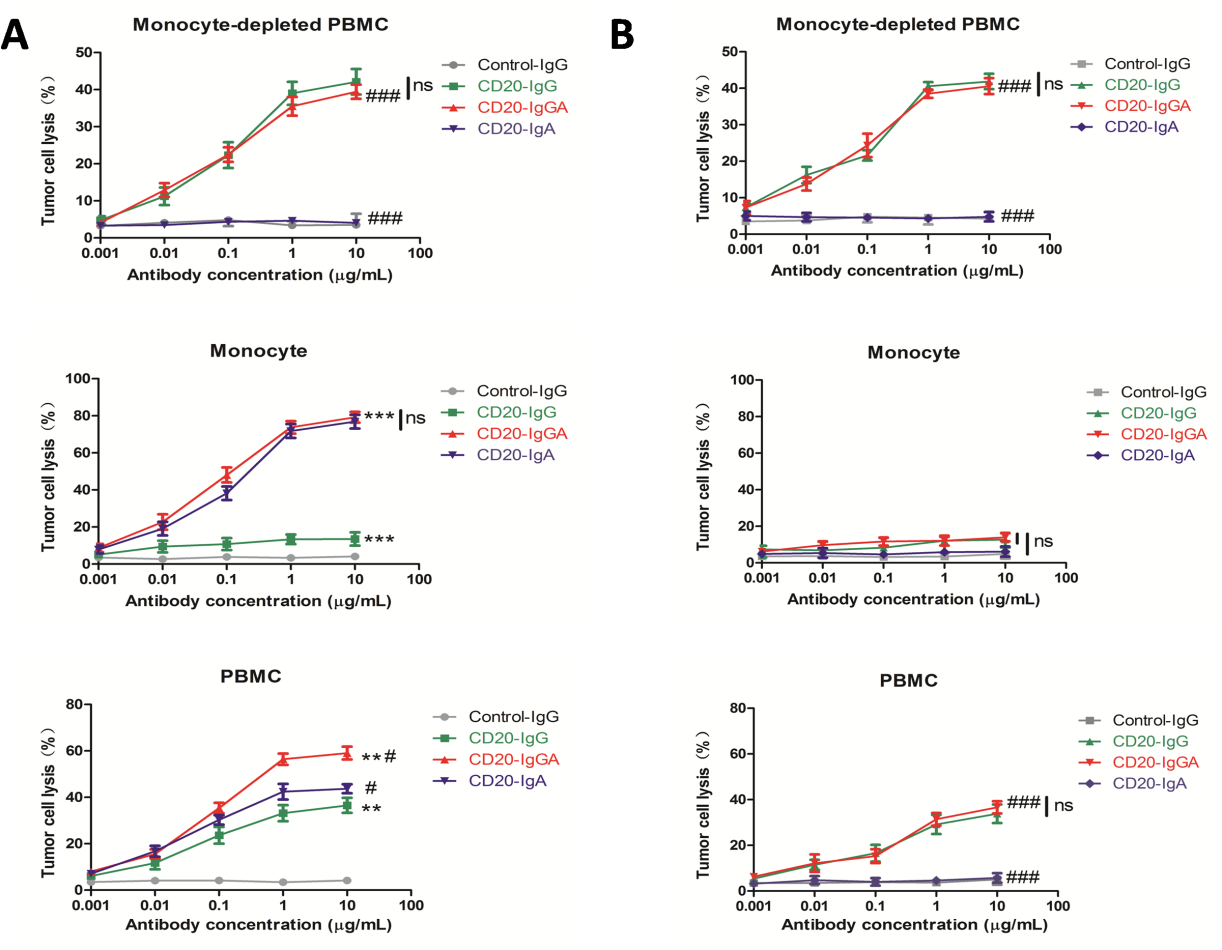
ADCC with different mouse effector cells *ex vivo* ADCC results are shown using Raji cells as targets and the mouse monocyte-depleted PBMC fraction, isolated CD14+monocytes, or PBMCs (E:T = 40:1) from FcaRI Tg mice **(A)** or wild-type C57BL/6 mice **(B)** in the presence of anti-CD20 antibodies. For monocytes and PBMCs, a 20-h ADCC assay was used. For monocyte-depleted PBMCs, a 4-h ADCC assay was used. All data are presented as the mean ± SEM (n = 3) from one of three representative experiments. *CD20-IgGA group versus CD20-IgG group; #CD20-IgGA group versus CD20-IgA group. **P < 0.01; ***P < 0.001; #*P* < 0.05; ###*P* < 0.001; ns, not statistically significant by two-way ANOVA.

To investigate whether anti-CD20 antibodies mediate antitumor activity by mouse blood effector cells, we generated a human FcαRI (CD89) Tg mouse strain using an authentic murine CD14 promoter with CD89 expression restricted to blood and tissue monocytes/macrophages [4]. A previous study showed that monocytes/macrophages mediate ADCC in an overnight assay [2]. Therefore, we used isolated CD14-positive monocytes from FcαRI Tg mice as effector cells in a 20-h assay. CD20-IgGA and CD20-IgA mediated potent tumor lysis, which exceeded tumor lysis mediated by CD20-IgG (Figure 1A). Wild-type (WT) monocytes were not able to lyse target cells using CD20-IgA, while tumor lysis by CD20-IgGA was similar to that mediated by CD20-IgG (Figure 1B), indicating that the cytotoxic activity of IgA Fc *ex vivo* was fully dependent on the presence of FcαRI. Monocyte-depleted PBMCs were able to efficiently lyse target cell utilizing both CD20-IgG and CD20-IgGA, through mechanisms likely mediated by NK cells, indicating that NK-mediated ADCC activity could be induced by CD20-IgGA (Figure 1A). As expected, CD20-IgGA exhibited greater cytotoxicity than CD20-IgG and CD20-IgA using PBMCs from Tg mice (which include monocytes and NK cells) as effector cells against Raji cells in an overnight assay. Because mouse PMNs do not express FcγRIa or FcγRIIIb [2], mouse PMN assays were not carried out.

Taken together, these results showed that CD20-IgGA was more efficient than CD20-IgG or CD20-IgA in tumor cell killing by both human myeloid effector cells and Tg mouse PBMCs.

### Macrophage-mediated ADCP induced by anti-CD20 antibodies *in vitro*

**Figure 2:**
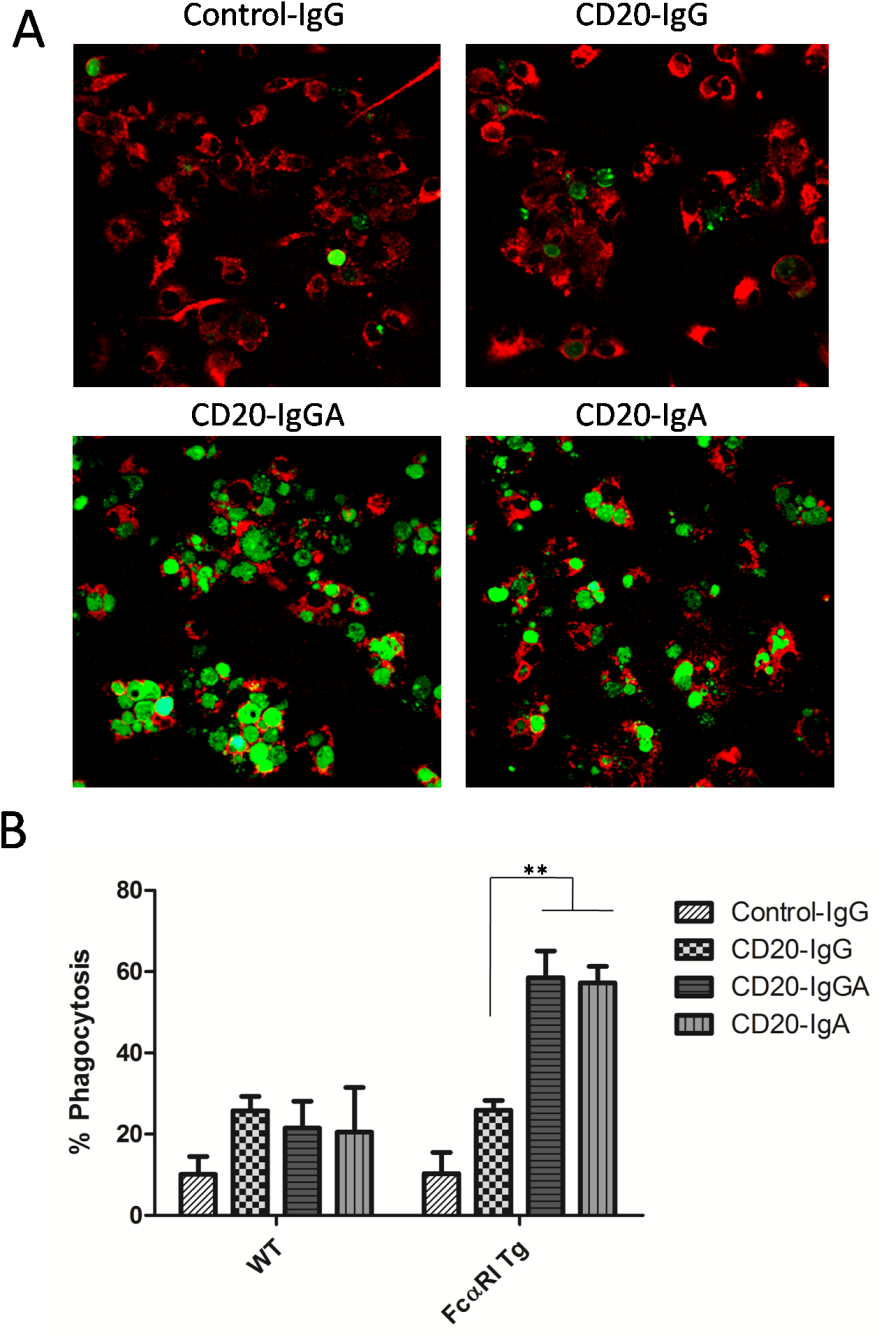
ADCP assays with mouse BMDMs *In vitro* assay to determine elimination of Raji cells by macrophages. **(A)** BMDMs from WT and FcαRI Tg C57BL/6 mice were incubated with CFSE-labeled Raji cells at an E:T ratio of 5:1 in the presence of 10 μg/mL anti-CD20 antibodies. After a 4-h incubation, cells were transferred to a new tube and visualized via confocal microscopy. Representative images of ADCP mediated by anti-CD20 antibodies are shown. **(B)** BMDMs were labeled with APC-F4/80 antibodies and cocultured with CFSE-labeled Raji cells in the presence of the indicated antibodies. Phagocytosis of Raji cells was analyzed by FACS and quantified as the percentage of double-positive cells relative to total CFSE-positive cells and F4/80+cells. **P < 0.01 using unpaired two-tailed t tests.

Next, we investigated whether macrophages could be recruited by anti-CD20 antibodies to mediate ADCP against Raji cells. Macrophages express high levels of FcαRI/FcγRs and can eliminate pathogenic cells primarily via phagocytosis [2]. Bone marrow-derived macrophages (BMDMs) of FcαRI Tg and WT C57BL/6 mice were used to evaluate the ability of anti-CD20 antibodies to facilitate ADCP. After a 4-h incubation in the presence of anti-CD20 antibodies, phagocytosis of carboxyfluorescein succinimidyl ester (CFSE)-Raji cells by macrophages was observed by confocal microscopy (Figure 2A). Phagocytosis was also evaluated by fluorescence-activated cell sorting (FACS); macrophages were labeled with APC-conjugated anti-mouse F4/80 antibodies and incubated with target cells labeled with CFSE in the presence of anti-CD20 antibodies. A PE anti-CD19 antibody was also used as the control to discriminate between target cell adhesion and phagocytosis. Phagocytosis was reported as the fraction of triple-positive cells relative to the total number of tumor cells in the sample (Figure 2B). CD20-IgGA and CD20-IgA were able to effectively clear up to 60% of the targeted tumor cells, a much higher degree of phagocytosis than observed with CD20-IgG. The results shown in Figure 2A mirrored those of ADCP assays analyzed by FACS. Only the IgA2-Fc-containing formats, CD20-IgA and CD20-IgGA, exhibited obvious phagocytosis compared with the CD20-IgG and control IgG. These observations were consistent with those of previous studies [2].

### Pharmacokinetics (PK) analysis of anti-CD20 antibodies in C57BL/6 mice

**Figure 3:**
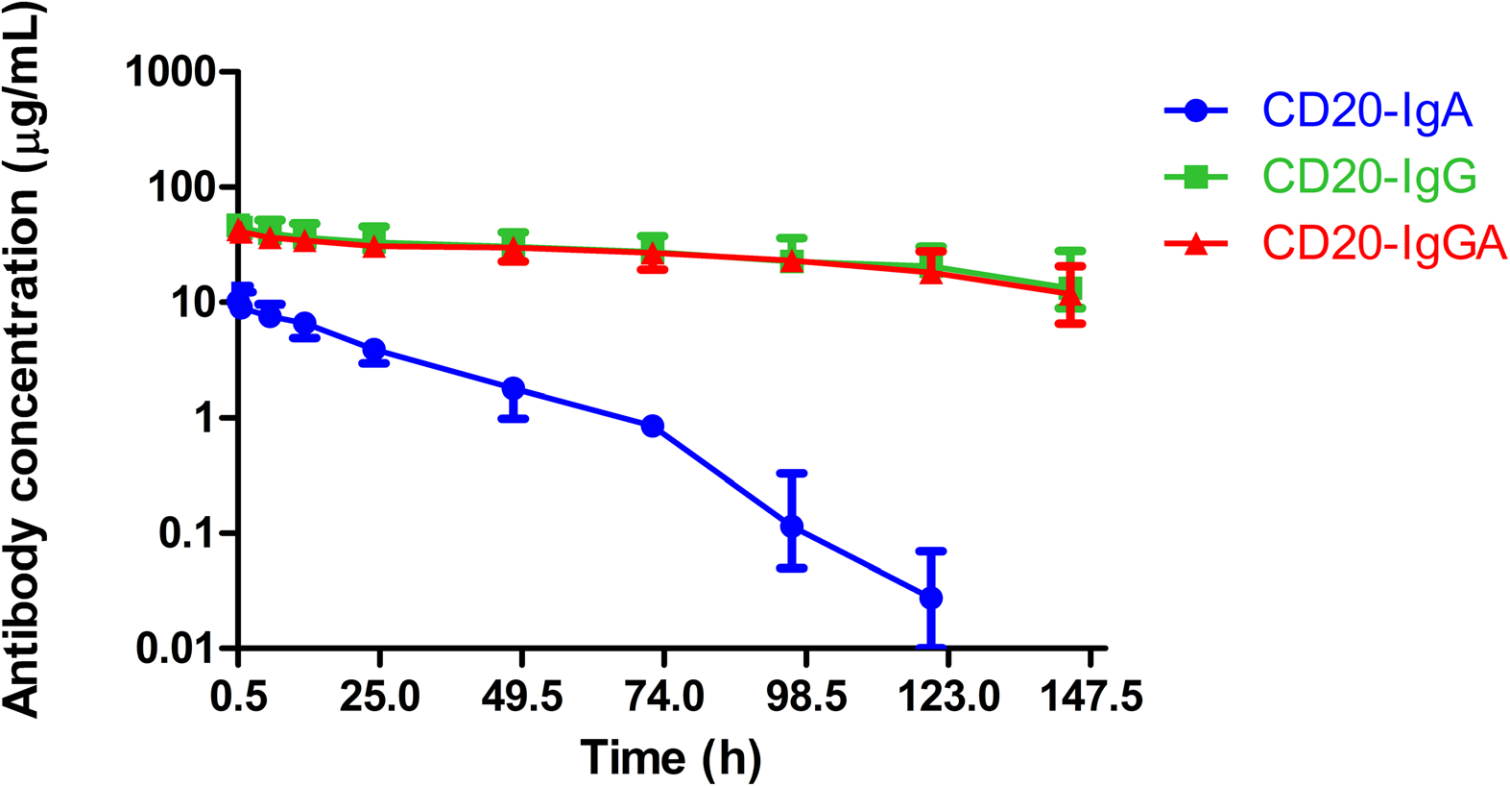
Serum half-life of anti-CD20 antibodies in C57BL/6 mice Serum levels for CD20-IgG (green), CD20-IgGA (red), and CD20-IgA (blue) in C57BL/6 mice (n = 6/group) were quantified by ELISA for detection of human IgG and IgA. Mice were intravenously (i.v.) injected with 100 μg anti-CD20 antibodies, and blood samples were collected at the indicated time points.

Human IgA antibodies do not bind to FcRn; therefore, the half-life of mouse or human IgA in mice is much shorter than that of human IgG, estimated to be in the range of 12–48 h [2]. Because CD20-IgGA contains both IgG Fc and IgA Fc, the IgGA antibody is expected to be recycled efficiently by FcRn, resulting in IgG1-like serum persistence (Supplementary Table 1). To determine the serum half-life of anti-CD20 antibodies in C57BL/6 mice, we injected 100 μg CD20-IgGA, CD20-IgG, or CD20-IgA intravenously (i.v.) and measured the serum antibody concentration at the indicated time points (Figure 3). As expected, CD20-IgGA exhibited a clearance rate similar to that of the parental IgG1 molecule, with half-lives of 4.2 and 4.4 days (Table 1), respectively, suggesting that FcRn recycling was not influenced by the IgA Fc region. In contrast, CD20-IgA exhibited rapid clearance, showing a substantially shorter serum half-life in mice than IgGA and IgG (half-life of 10.3 h; Table 1). These findings were consistent with a previously published study [5].

**Table 1:** Parameter of pharmacokinetics

Parameter	CD20-IgG	CD20-IgGA	CD20-IgA
t1/2 (h)	106.54	100.57	10.33
CL(L/h)	14.96	17.9	36.88
MRT (h)	147.92	136.43	24.05

t1/2: Elimination Half-life

CL: clearance

MRT: mean residence time.

### Comparison of the *in vivo* antitumor activities of three types of anti-CD20 antibodies

Because CD20-IgGA and CD20-IgA exhibited stronger ADCC and ADCP than CD20-IgG *in vitro* with both human and mouse effector cells, we next performed *in vivo* studies to assess the antitumor activities of the panel of anti-CD20 antibodies. To study the ADCC and ADCP effects mediated by IgG- and IgA-Fc domains in the C57BL/6 background, LLC-CD20 mice were generated using the mouse LLC cell line, which was transduced with a human *CD20* gene to facilitate tumor growth in C57BL/6 mice. Because CD20-transfected LLC cells lacked signaling pathways related to cell growth and cell apoptosis, Fab-dependent effector mechanisms did not contribute to the antitumor effects in this model, which allowed us to focus on Fc-dependent killing.

**Figure 4:**
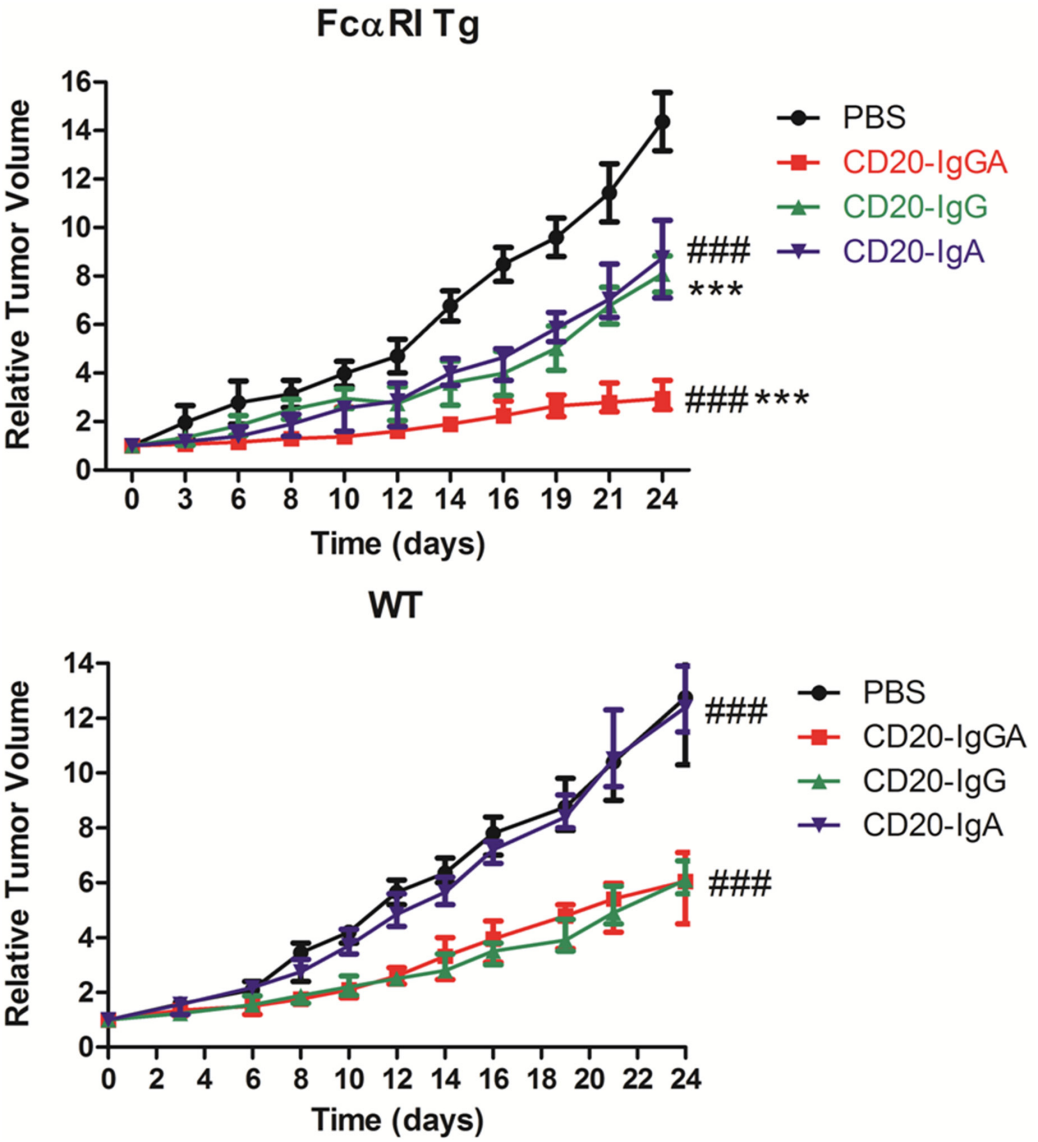
Comparison of the efficacy of anti-CD20 antibodies in the LLC-CD20 xenograft model FcαRI Tg **(A)** or WT **(B)** C57BL/6 mice were injected s.c. with 1 × 10^6^ LLC-CD20 cells. Mice bearing xenografts (tumor size ~100–150 mm^3^) were administered 5 mg/kg CD20-IgG and CD20-IgGA (n = 6–8/group) via i.v. injection on days 0, 4, 8, 12, 16, and 20 or 5 mg/kg CD20-IgA daily from day 0 to day 23. *CD20-IgGA-treated group versus CD20-IgG-treated group; #CD20-IgGA-treated group versus CD20-IgA-treated group. ***P < 0.001 and ###*P* < 0.001 using two-way ANOVA.

Mice were inoculated with LLC-CD20 cells and treated with CD20-IgGA, CD20-IgG, or CD20-IgA. Treatment of tumor-bearing mice with CD20-IgGA induced significant and persistent tumor regression in Tg mice; this antitumor activity was better than that of CD20-IgG and CD20-IgA (Figure 4A). Thus, the IgA Fc exerted greater antitumor activities than IgG Fc in the Tg model. In contrast, no differences were observed between CD20-IgGA and IgG treatment, and CD20-IgA treatment had almost no effect in WT mice (Figure 4B), indicating that *in vivo* antitumor activity was strictly dependent on the presence of FcαRI.

### Macrophage-mediated anti-CD20 antibody activity in an intraperitoneal model

**Figure 5:**
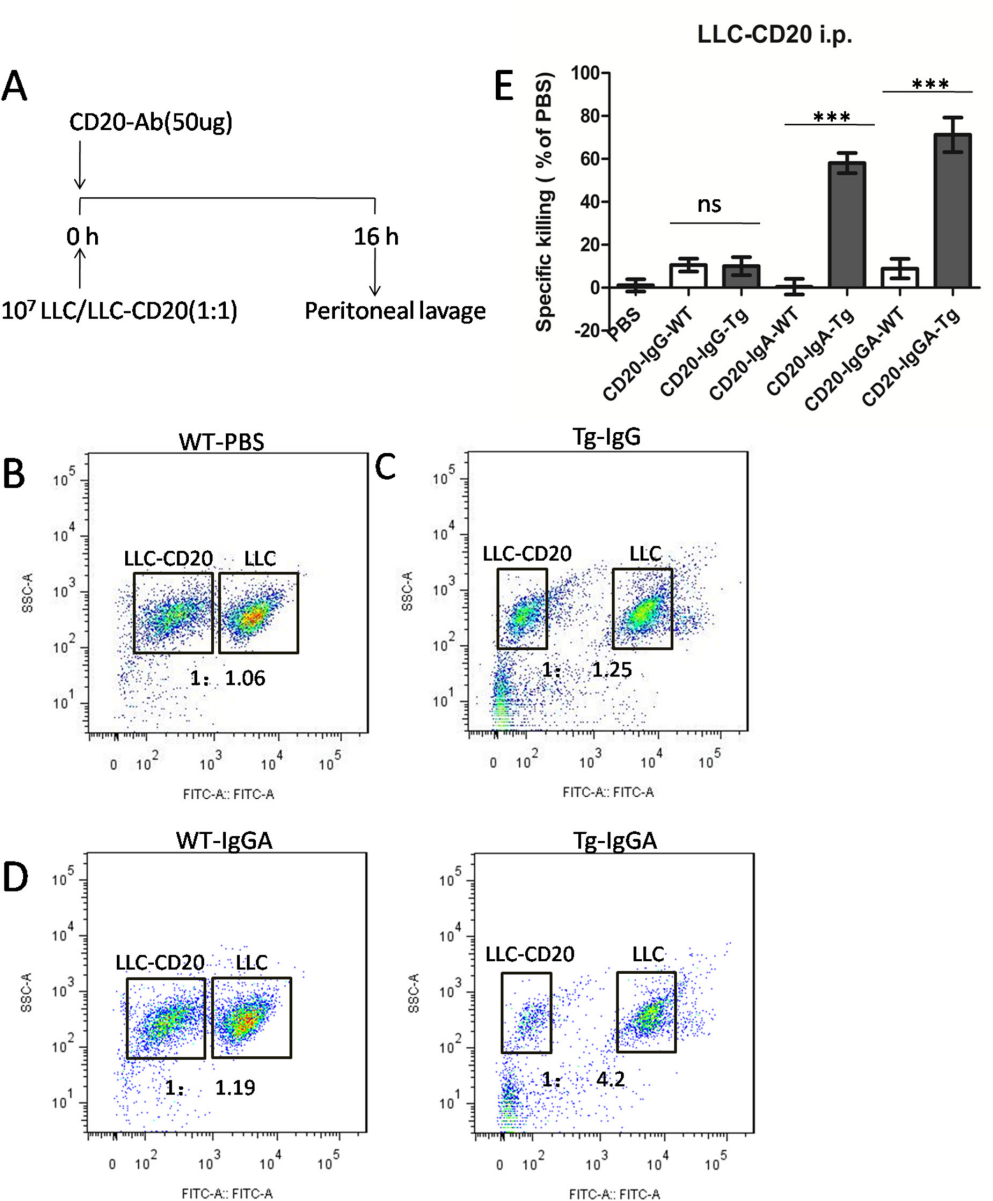
Macrophages mediated *in vivo* anti-CD20 antibody activity in a peritoneal model using LLC-CD20 cells **(A)** The LLC-CD20 i.p. model was generated by injection of a 1:1 mixture of LLC and LLC-CD20 cells (1 × 10^7^ cells) differentially labeled with high and low concentrations of CFSE into the peritoneum of FcαRI Tg or WT BALB/c mice (n = 6/group). Immediately after injection of the cells, the mice received 50 μg anti-CD20 antibodies. After 16 h, the peritoneum was washed, and the ratios of CSFE+LLC to CSFE++LLC-CD20 cells were analyzed to calculate specific cytotoxicity. **(B–D)** Representative dot plots showing cells retrieved from the peritoneum. LLC and LLC-CD20 cells could be identified based on CFSE labeling. **(E)** Specific cytotoxicity was calculated from the ratio of LLC to LLC-CD20 cells in each treatment group. ***P < 0.001; ns, not statistically significant using unpaired two-tailed t tests.

Next, we used a peritoneal model to analyze effector-target interactions and antitumor mechanisms of antibodies (Figure 5A), as previously reported [2]. Our results showed that CD20-IgG treatment did not induce significant killing of LLC cells (Figure 5C). However, CD20-IgGA and CD20-IgA still showed significant cytotoxicity in this intraperitoneal model in the FcαRI Tg background. In WT mice, IgGA and IgA were not effective, indicating that the Fab region did not contribute to the antitumor activity under these conditions (Figure 5D). Effector cells in the peritoneum include macrophages, resident monocytes, and PMNs. Thus, it is likely that peritoneal macrophages and monocytes were the main effector cells responsible for the cytotoxicity induced by IgA Fc in this model because PMNs do not express mouse FcγRs and human FcαRI.

Taken together, these findings suggested that IgA Fc mediated efficient *in vivo* cytotoxicity of LLC-CD20 cells in a short-term peritoneal model and that these effects were fully dependent on the IgA Fc interaction with FcαRI on macrophages.

## DISCUSSION

Currently, all of the therapeutic antibodies approved by the FDA are IgG antibodies. IgG can induce NK cell-mediated ADCC, CDC, and apoptosis *in vivo*. Although IgG can efficiently activate FcγRs (FcγRIIIa and FcγRIIa) and induce ADCC, IgG may interact with inhibitory FcγRIIb and FcγRIIIb on several types of effector cells, thereby weakening the cytotoxicity of effector cells [2]. IgA is the most abundantly expressed immunoglobulin on mucosal surfaces and exhibits antimicrobial effects [2]. Additionally, IgA can activate effector cells to stimulate a respiratory burst, phagocytosis, antigen presentation, and ADCC by binding to CD89 [6]. However, IgA antibody therapy faces development changes due problems with aggregation and a shorter half-life versus IgG antibodies [2]. Therefore, IgG/IgA “cross-isotype” molecules may be benefitted by providing advantages of both IgG and IgA [2]. Moreover, some studies have shown that FcγR pathways and functions do not overlap with those of FcαRI on the same effector cells [2]. In an *in vitro* study, monocytes, which co-express FcγRs and FcαRI, were found to display increased cytotoxicity with a combination of IgG and IgA antibodies in an overnight killing assay [7].

In this study, we constructed hybrid CD20-IgG/IgA molecules having the structure of CD20 IgG-linker-IgACH_2_CH_3_. The IgGA construct exhibited an enhanced ability to induce ADCC by human blood cells by engaging cytotoxic PMNs and NK cells. Importantly, CD20-IgGA had a half-life similar to that of IgG (about 5 days), whereas CD20-IgA had a half-life of only 16.8 h, consistent with published studies showing a half-life of 12–48 h [7]. IgGA Fc also retained C1q binding (Supplementary Table 1) and exhibited CDC activity against CD20-expressing Raji cells (data not shown). These results showed that the hybrid IgG/IgA molecules possessed the advantages of both IgG and IgA antibody types.

The hybrid IgG/IgA construct exhibited antitumor activity in a unique Tg mouse model expressing CD89 controlled by the murine CD14 promotor, restricting expression to monocytes, macrophages, and Kupffer cells. In addition to PMNs and NK cells, monocytes and macrophages have been shown to function as potent effector cells in ADCC and phagocytosis [2]. Although monocytes and macrophages express both IgG and IgA FcR, CD20-IgG/IgA and CD20-IgA showed better ADCC and ADCP effects than IgG *in vitro*, and CD20-IgG/IgA showed significant antitumor effects *in vivo* using LLC-CD20 cells in the Tg-C57BL/6 model. Thus, the mononuclear phagocyte network was the predominant effector cell population *in vivo*.

Despite the absence of PMN-mediated cytotoxicity, IgGA could facilitate ADCC and ADCP from both NK cells and monocytes/macrophages, consistent with a previous study in which direct activation of macrophages by IgA was demonstrated *in vivo* [2]. Boross *et al* provided evidence that in CD89 Tg mice, IgA antibodies could induce phagocytosis of tumor cells by macrophages in a short-term peritoneal model [2]. Similarly, CD20-IgGA and IgA also showed significant cytotoxicity in our intraperitoneal model in the FcαRI Tg background.

Although our model may not mimic the situation in humans because of the restriction of CD89 expression to monocytes and macrophages, our findings of the activation of monocytes and macrophages are important, particularly considering the abundant infiltration of tumor-associated macrophages in solid tumors. Future studies are needed to explore the possibility of using CD89 as a potent activator of PMNs, monocytes, and macrophages to augment antibody-mediated antitumor therapy.

## MATERIALS AND METHODS

### Mice

Human FcaRI Tg mice were generated with the murine CD14 promoter controlling the expression of CD89, as previously described [4]. A knock-in targeting vector was designed that contained a cDNA encoding the human *CD89* cDNA, the 2A self-processing peptide, and 5.2 kb of a sequence homologous to the murine *CD14* gene. Homologous recombinant embryonic stem (ES) cells were micro-injected into C57BL/6 blastocysts. The resulting chimeric mice were crossed with C57BL/6 mice, and a stable line was established in the C57BL/6 background. Littermates not expressing the human *CD89* gene were used as controls.

All animal experiments were approved by the Animal Ethics Committee of Tongji University and were in accordance with the Association for Research in Vision and Ophthalmology (ARVO) statement for the use of Animals in Ophthalmic and Vision Research. C57BL/6 mice were housed in a pathogen-free animal facility at the experimental animal center of Tongji University. Mice were fed standard chow and provided with distilled water *ad libitum*. Cages were refreshed weekly. Mice were euthanized using sodium pentobarbital, and appropriate efforts were made to minimize animal suffering.

### Cell lines

Raji and Lewis lung cancer (LLC) cell lines (ATCC) were cultured in RPMI1640 or Dulbecco's modified Eagle's medium (DMEM; Gibco) supplemented with 10% fetal bovine serum (FBS). LLC cells were transfected with the human *CD20* gene (Addgene), and CD20-expressing clones were selected using cytofluorimetry.

### Antibody expression and purification

Rituximab (accession number: DB00073) was used as the CD20-IgG control in this paper. The CD20-IgGA used in this study was generated by fusing human IgA2-CH_2_-CH_3_ to the C-terminus of rituximab with a (G_4_S)_3_ linker. The variable region of the HC and LC for the generation of CD20-IgA was derived from rituximab. All antibodies were transiently expressed in HEK293F cells in Freestyle medium (Life Technologies). At 5–6 days after transfection, the cell suspension was centrifuged for purification. IgG and IgGA antibodies were purified by standard protein A affinity chromatography (Life Technologies). IgA2 was purified via Peptide M agarose resin (Invivogen). Samples were buffer-exchanged into 1× phosphate-buffered saline (PBS) using Amicon Ultra-4 (Millipore) spin columns with a 30-kDa cutoff, and the purity of purified samples was assessed by sodium dodecyl sulfate polyacrylamide gel electrophoresis (SDS-PAGE) using 10% gradient gels.

### ADCC assay

CytoTox96 nonradioactive assays (Promega) were used to evaluate the capacity of mouse blood cells to trigger lysis of tumor cells. PBMCs were isolated from freshly drawn peripheral blood of CD89 Tg and WT mice using mouse Percoll (Sigma) following the manufacturer's directions. Monocytes were isolated from the PBMC fraction using CD14-positive microbeads (Miltenyi Biotech). PBMCs without monocytes were used as effector cells. Effector cells were plated (8 × 10^4^cells/well) in 96-well plates. Raji cells (2 × 10^3^ cells/well) were also transferred to the same plates, and cultures were incubated with recombinant anti-CD20 antibodies at different concentrations (0.001, 0.01, 0.1, 1.0, or 10.0 μg/mL) at 37°C for 4 h for monocyte-depleted PBMC assays or 20 h for monocytes and PBMCs. An IgG isotype (Abcam) was used as a negative control. The lactate dehydrogenase (LDH) released into the medium following challenge with anti-CD20 antibodies was quantified by measuring the absorbance at 490 nm. The LDH activity in supernatants from cocultures of effector cells and Raji cells, effector cells cultured in the absence of the cell lysis target, and Raji cells cultured in the absence of effector cells was used to calculate the corrected experimental, effector, and target values, respectively. These values were then used to calculate cytotoxicity expressed as a percentage of the target cells according to routine procedures.

### ADCP analysis

BMDMs of mice were isolated and cultured as previously described [8]. Macrophages were seeded at 5 × 10^4^ cells/well in 96-well plates, and CSFE-Raji cells (1 × 10^4^cells/well; 10 mM; Life Technologies) were also transferred to the same plates and were incubated with 5.0 μg/mL anti-CD20 antibodies at 37°C. After 4 h, adherent cells were detached and stained with APC-conjugated anti-mouse F4/80 antibody (F4/80-APC; BD Pharmingen). The PE-conjugated anti-human CD19 antibody (CD19-PE; BD Pharmingen) was used as a control to discriminate between target cell adhesion and phagocytosis. Phagocytosis was evaluated by FACS on a FACSVerse (BD Bioscience) and reported as the fraction of triple-positive cells over the total number of tumor cells in the sample.

For microscopy experiments, macrophages and CFSE-Raji cells were incubated on cover slips in 24-well plates at the appropriate effector tumor cell ratio (5:1) along with 5.0 μg/mL anti-CD20 antibodies at 37°C for 4 h. Subsequently, cells were fixed, followed by permeabilization, staining of macrophages using a rabbit anti-mouse F4/80 antibody and a goat anti-rabbit IgG-Cy3 antibody (Boster Biotech, Wuhan, China), nuclear staining by 4′,6-diamidino-2-phenylindole (DAPI; Boster Biotech), and confocal microscopy according to routine procedures.

### PK study

IgG1 CD20, Ig1/IgA2 CD20, or IgA2 CD20 (100 μg/mouse) was injected intravenously into C57BL/6 mice (four mice/group). Blood was collected via the tail vein from alternating mice at the indicated time points. The human IgA antibody concentrations in the sera were determined by enzyme-linked immunosorbent assays (ELISAs) using human IgA ELISA kits (Bethyl Laboratories), and human IgG and IgGA antibody concentrations in the sera were determined using human IgG ELISA Kits (Bethyl Laboratories) according to the manufacturer's instructions. The data were analyzed by PK solver software, as previously described [2].

### LLC-CD20 models

LLC cells were subjected to lentivirus-mediated transduction of human CD20. A clone was established that reproducibly grew in WT C57BL/6 mice. LLC-CD20 cells (1 × 10^6^) were subcutaneously (s.c.) injected into WT and FcαRI Tg C57BL/6 mice. Animals were randomly assigned into treatment groups (6–8 per group), with the mean tumor volume for each group being 100–150 mm^3^. Tumor size was monitored twice a week, and tumor volumes were determined according to the formula: tumor volume (mm^3^) = longer diameter × (shorter diameter)^2^ × 0.5. PBS was administered as the vehicle control for all groups. IgG-CD20 and IgGA-CD20 (5 mg/kg) were administered intravenously (i.v.) twice a week for 3 weeks (days 0, 4, 8, 12, 16, and 20). CD20- IgA (5 mg/kg) was administered i.v. daily beginning from day 0 to day 23.

### LLC peritoneal model

The LLC peritoneal model was established as previously described [9]. LLC and LLC-CD20 cells were labeled with 0.1 μM and 1 μM CFSE (Life Technologies) and mixed at a 1:1 ratio. Next, 10^7^ cells were intraperitoneally injected into WT and FcαRI Tg C57BL/6 mice (six per group). Anti-CD20 antibodies (50 μg/mouse) were injected intraperitoneally into mice directly after injection of tumor cells. Sixteen hours later, the mice were euthanized, and the peritoneum was washed. The ratio of LLC cells (CSFE+) to LLC-CD20 cells (CSFE++) was determined by cytofluorimetry.

### Statistical analysis

Data were graphed and analyzed using GraphPad Prism 6.0 (Graph Pad Software). Data are expressed as the median and range or mean ± standard error of the mean (SEM). Quantitative data between groups were compared using Student's t test with unpaired two-tailed t tests or two-way analysis of variance (ANOVA). Differences with *P* values of less than 0.05 were considered statistically significant.

## SUPPLEMENTARY MATERIALS FIGURES AND TABLES



## Abbreviations

NHL: non-Hodgkin's lymphoma
FDA: US Food and Drug Administration
ADCC: antibody-dependent cell-mediated cytotoxicity
CDC: complement-mediated cytotoxicity
PMN: polymorphonuclear leukocyte
Tg: transgenic
NK: natural killer
ADCP: antibody-dependent cell-mediated phagocytosis
EGFR: epidermal growth factor receptor
ES: embryonic stem
LLC: Lewis lung cancer
FBS: fetal bovine serum
DMEM: Dulbecco's modified Eagle's medium
SDS-PAGE: sodium dodecyl sulfate polyacrylamide gel electrophoresis
PBS: phosphate-buffered saline
PBMCs: peripheral blood mononuclear cells
WT: wild-type
LDH: lactate dehydrogenase
BMDM: bone marrow-derived macrophage
PK: pharmacokinetics
CFSE: carboxyfluorescein succinimidyl ester.
