# Supplementary material for: Simultaneous exposure to FcγR and FcαR on monocytes and macrophages enhances antitumor activity in vivo
Source: Oncotarget. 2017 Apr 10;8(24):39356–66. doi: 10.18632/oncotarget.17000 (PMC5503618; doi:10.18632/oncotarget.17000)
Supplement: Supplementary file 1 [file oncotarget-08-39356-s001.pdf]

Simultaneous exposure to FcγR and FcαR on monocytes and macrophages enhances antitumor activity *in vivo*

SUPPLEMENTARY DATA

SUPPLEMENTARY EXPERIMENTAL PROCEDURES

In vitro binding activity assay

The binding of CD20, FcαRI, FcγR, C1q, and human FcRn (hFcRn) to CD20-IgGA, CD20-IgG, and CD20-IgA was monitored by an in vitro binding activity assay. Specifically, we first coated 96-well plates overnight with CD20/MS4A1 protein (Sino Biological), recombinant human FcαRI (Sino Biological), recombinant human FcγRIIIa-V158 (Novoprotein), C1q (Quidel), and recombinant human FcRn (Novoprotein). The next day, the 96-well plates were rinsed with PBS, and titrated CD20 mAbs were added to each well. The plates were incubated at 37°C for 1 h. After rinsing in PBS, the plates were incubated with an anti-human IgG Fc antibody conjugated with horseradish peroxidase (HRP; Bethyl Laboratories) at 37°C for 1 h. Next, the plates were rinsed in PBS and incubated briefly with TMB peroxidase substrate (KPL, Gaithersburg). The absorbance rates were then measured using a microplate reader (Molecular Devices, USA), the values were used for analysis with GraphPad Prism 6.0 (GraphPad Software). For the calculation of KDs, the equation for one-site-binding(hyperbolic) was used.

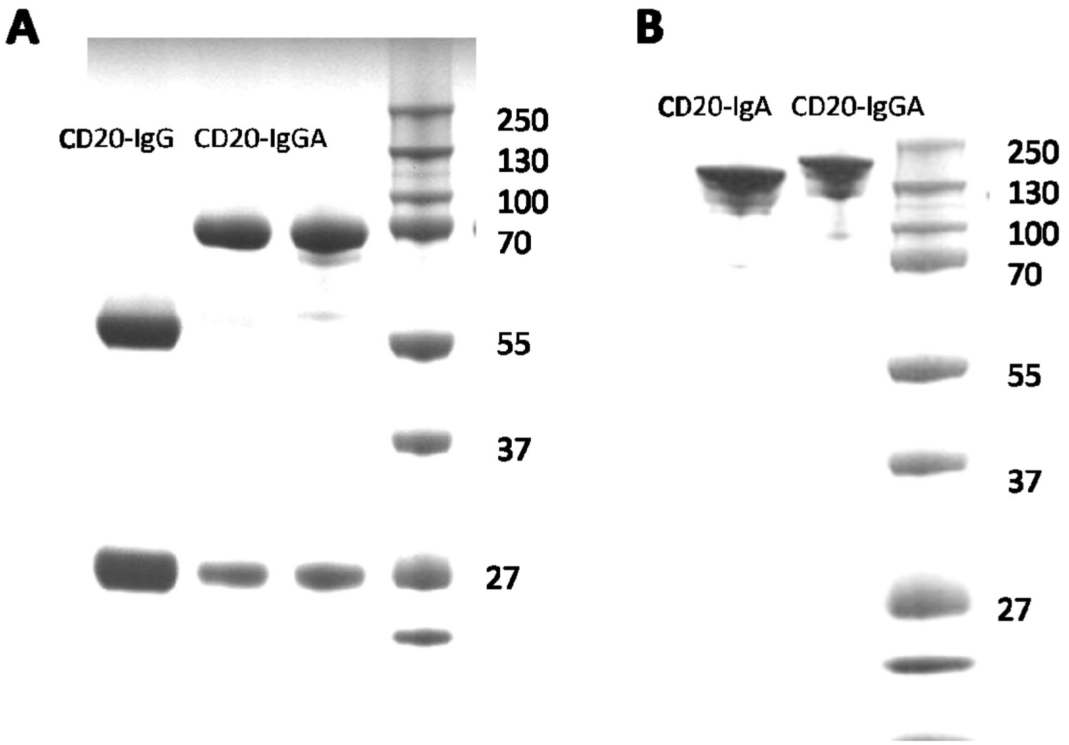

Supplementary Figure 1: Expression of CD20-IgGA, CD20-IgA, and CD20-IgG in HEK293F cells. Antibodies were expressed in HEK293F cells, purified by Peptide M agarose resin (CD20-IgA) or Protein A chromatography (CD20-IgG, CD20-IgGA), and separated by **(A)** reducing and **(B)** non-reducing SDS-PAGE on 10% gels.

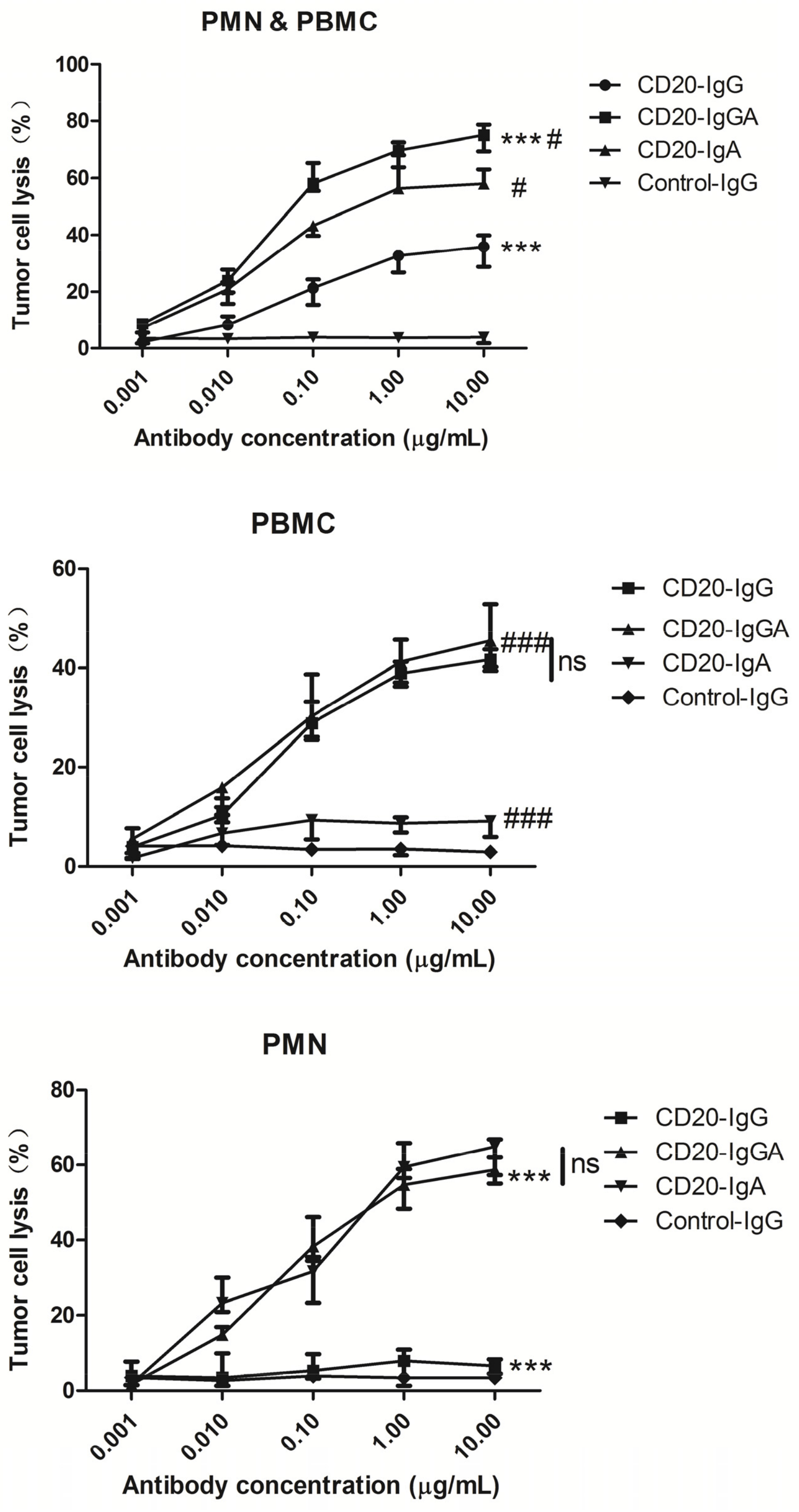

Supplementary Figure 2: ADCC results are shown using Raji cells as targets with different effector cell types (freshly isolated PBMCs, PMNs, or both PMNs and PBMCs combined, E:T = 40:1). Data are the average of three different experiments with three separate donors. *CD20-IgGA group versus CD20-IgG group; #CD20-IgGA group versus CD20-IgA group. ***$P < 0.001$; #$P < 0.05$; ###$P < 0.001$; ns, not statistically significant by two-way ANOVA.

Supplementary Table 1: CD20, FcαRI, FcγR, FcRn, and C1q binding of antibodies determined by ELISA

	K_D values (nM)				
	**CD20**	**FcαRI**	**FcγRIIIa**	**hFcRn**	**C1q**
CD20-IgG	8.9	N.B.	512	1015	256
CD20-IgGA	9.3	280	488	1360	577
CD20-IgA	6.0	322	N.B.	N.B.	N.B.

N.B. indicates no detectable binding.
